# Ab Initio Calculation of Energy Gap and Optical Gap of Organic Semiconductors PTCDA and PDI

**DOI:** 10.1002/cphc.202500481

**Published:** 2026-02-10

**Authors:** Chieh‐Min Hsieh, Tanja Link, Michael Maas, Katharina Koschek, Tim Neudecker

**Affiliations:** ^1^ University of Bremen Institute for Physical and Theoretical Chemistry Bremen Germany; ^2^ Advanced Ceramics Group University of Bremen Bremen Germany; ^3^ Fraunhofer IFAM Bremen Germany; ^4^ MAPEX Center for Materials and Processes Bremen Germany; ^5^ Bremen Center for Computational Materials Science Bremen Germany

**Keywords:** crystal structure prediction, DFT, electronic properties, GW approximation, organic semiconductors

## Abstract

We applied ab initio methods, including the GW approximation, the Koopmans functionals, range‐separated hybrid functionals, and time‐dependent density functional theory (TDDFT) to investigate the energy gap and optical gap of perylene‐3,4,9,10‐tetracarboxylic dianhydride (PTCDA) and perylene diimide (PDI) derivatives. A detailed comparison of our calculated results from all the methods with experimental values was made, with particular focus on the properties of single molecules versus molecular crystals. Single‐molecule TDDFT with the polarizable continuum model (PCM) shows reasonable accuracy for the energy and optical gaps in molecular crystals but is less accurate in predicting ionization energy (IE) and electron affinity (EA) compared to GW calculations. These findings provide guidance for selecting reliable computational approaches for evaluating key electronic and optical properties of organic semiconductor systems.

## Graphical abstract

We benchmark electronic structure methods for predicting energy and optical gaps of three organic semiconductors from molecules to crystals. GW is most reliable for crystalline energy levels, while single‐molecule time‐dependent density functional theory with a polarizable continuum model delivers remarkable accuracy for some quantities at a much lower cost. The results offer practical guidance for efficient modelling of organic semiconductors.

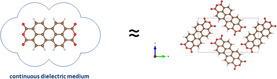

## 1 Introduction

**FIGURE 1:**
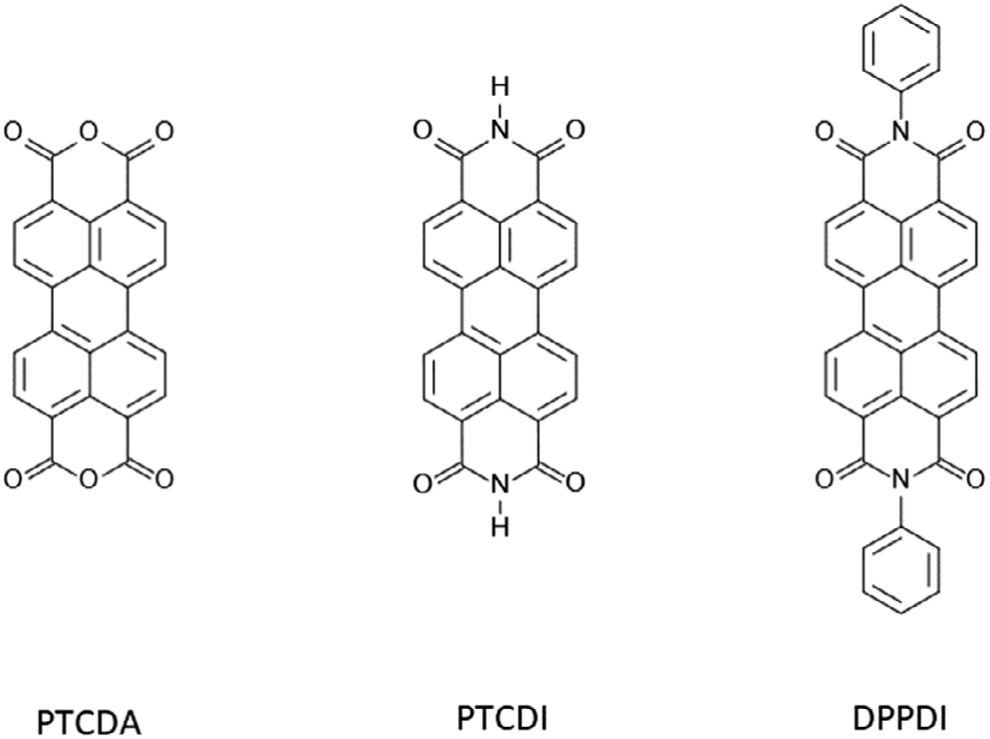
Molecular systems under investigation: perylene‐3,4,9,10‐tetracarboxylic dianhydride (PTCDA), perylene‐3,4,9,10‐tetracarboxylic diimide (PTCDI), and N,N′‐diphenyl‐3,4,9,10‐perylenedicarboximide (DPPDI).

Perylene‐3,4,9,10‐tetracarboxylic dianhydride (PTCDA) is an organic molecule consisting of a perylene core and carboxylic anhydride groups (Figure 1). Its *π*‐conjugated system features delocalized *π*‐electrons across the perylene framework, which stabilizes the molecule and enhances its unique electronic properties [1, 2]. PTCDA serves as a precursor for the synthesis of perylene diimide (PDI), which, like PTCDA, belongs to the rylene dye family. Moreover, PDIs can be chemically functionalized—such as at the imide positions or along the perylene backbone—to tailor their electronic properties [3]. In terms of applications, PTCDA and PDI are widely utilized as organic semiconductors [4, 5], including their use in sensors [6] and photovoltaics [7, 8].

Numerous theoretical and computational studies have previously examined PTCDA, PTCDI, and related perylene derivatives, establishing a substantial foundation for understanding their electronic and optical properties [9, 10, 11, 12]. Additional efforts have addressed these molecules at interfaces, particularly the adsorption of PTCDA on various substrates [13, 14, 15, 16, 17]. Most of these works employ a single theoretical framework, which limits direct assessment of the relative performance of different modern electronic‐structure approaches. To the best of our knowledge, a systematic cross‐method evaluation of rylene diimides across both molecular and crystalline environments has not been reported.

One of the most important electronic properties of semiconductors is the energy gap, which determines their electrical behavior. In molecules, this gap corresponds to the energy difference between the highest occupied molecular orbital (HOMO) and the lowest unoccupied molecular orbital (LUMO), also called the HOMO–LUMO gap or the fundamental gap. In molecular crystals, the molecular orbitals of neighboring molecules overlap, causing the energy levels to broaden into electronic bands. The width of energy bands depends on intermolecular interaction; the stronger the electronic coupling (i.e., orbital overlap), the wider the resulting bandwidth [18]. The energy gap in this context, known as the transport gap or band gap, represents the energy required to transfer an electron from the valence band to the conduction band. In this paper, we refer to the energy gap of molecular crystals as the transport gap for consistency. Since different types of energy gaps are often confused, Bredas provides a useful review clarifying these distinctions [19].

Another type of gap is the optical gap, which refers to the energy difference between the ground state and the first excited state induced by photon absorption. This gap is slightly smaller than the energy gap, with the difference corresponding to the exciton binding energy, which quantifies the binding of an exciton, an electron–hole pair. Notably, the exciton binding energy is more significant in organic semiconductors than in inorganic semiconductors due to more localized valence electrons and weaker electronic screening [20].

Experimentally, the energy gap can be determined using ultraviolet photoelectron spectroscopy (UPS) and inverse photoemission spectroscopy (IPES), which measure ionization energy (IE) and electron affinity (EA), respectively. The energy gap is then determined as the difference between the IE and EA values. The optical gap can be determined via a UV/Vis absorption spectrum using a Tauc plot [21], which analyzes the absorption coefficient as a function of photon energy to extract the material's optical gap.

Computationally, ground‐state density functional theory (DFT) [22, 23] provides access to HOMO and LUMO energies, thus enabling an estimate of the energy gap, while time‐dependent density functional theory (TDDFT) [24] yields the full UV/Vis spectrum, providing information about the optical gap. However, it is well known that DFT with semilocal functionals often underestimates energy gaps, as the Kohn–Sham eigenvalues do not represent true quasiparticle energies due to limitations in the exchange‐correlation (XC) functional [25]. Despite the fact that IE and EA can practically be obtained by adding or removing an electron from the molecular system, self‐interaction error can still contribute to inaccuracies [26, 27]. To address these limitations, a variety of theoretical approaches have been proposed, such as the GW approximation [28, 29], the Koopmans functionals [30, 31], and range‐separated hybrid (RSH) functionals [32].

Although these approaches improve upon semilocal DFT, each carries distinct assumptions and domains of applicability. RSH functionals offer well‐behaved frontier orbital energies for isolated molecules but require system‐dependent parameter tuning, which limits the consistency of computations; Koopmans‐corrected DFT reduces self‐interaction and includes orbital‐dependent static screening, yet does not capture the full nonlocal and dynamical many‐body polarization relevant in condensed phases; and GW calculations depend sensitively on the starting point and convergence parameters, particularly for absolute energy level alignment.

In this paper, we applied these methods to investigate the energy gap of three molecular systems, i.e., PTCDA, perylene‐3,4,9,10‐tetracarboxylic diimide (PTCDI), and N,N′‐diphenyl‐3,4,9,10‐perylenedicarboximide (DPPDI), as illustrated in Figure 1. To this end, accurate prediction of these electronic and optical properties requires reliable structural information for the corresponding molecular crystals. When experimental crystal structures are unavailable, crystal structure prediction (CSP) [33] can in principle be used to identify low‐energy packing motifs. However, CSP accuracy varies for functionalized PDI derivatives and often requires extensive sampling. For this reason, we also considered whether a simpler continuum approach, the polarizable continuum model (PCM) [34], could approximate the crystalline environment. Here, we focus primarily on evaluating the electronic and optical properties using experimental crystal structures and PCM‐based models, while additional CSP attempts are provided in the Supporting Information.

## 2 Computational Details and Experimental Procedures

This section describes the computational methods used in our calculations, including a brief explanation of the underlying theories. Additionally, we present the experimental procedures for material synthesis and measurement, intending to provide a comprehensive overview of the methodologies applied in this study.

### 2.1 Crystal Structures

We used the crystal structures of PTCDA (*α*‐ and *β*‐phase) and PTCDI, characterized by Tojo and Mizuguchi [35, 36, 37], as well as the DPPDI crystal structure synthesized by Sato and Mizuguchi [38]. These structures were used exactly as reported in the experimental CIF files, with the original lattice parameters and atomic positions preserved. No geometry relaxation was applied prior to the GW calculations.

### 2.2 Range Separated Hybrid (RSH) DFT

RSH DFT with the LRC‐*ω*PBE functional [39], as implemented in Q‐Chem 6.0 [40], was used to calculate IE and EA. This approach combines GGA functionals for short‐range Coulomb interaction and Hartree‐Fock (HF) exchange for long‐range interactions. The calculations were carried out using the may‐cc‐pVDZ [41] basis set. Besides, the range separation parameter *ω* was optimized to ensure:
(1)

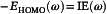



(2)

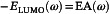

where *E*_HOMO_ and *E*_LUMO_ represent the orbital energies of HOMO and LUMO, while IE and EA were calculated as the total energy difference of the system upon removal or addition of an electron:

(3) IE=E(N)−E(N−1)

(4) EA=E(N+1)−E(N)

with *N* representing the number of electrons in the neutral system. This process was automated using the script “OptOmegaIPEA.pl,” which is included in the Q‐Chem package. For molecular crystals, the conductor‐like PCM (C‐PCM) model [42] was used to account for polarization effects. Although PCM is typically applied to simulate solvent effects, it has also been employed to model molecular crystals [43]. For this purpose, the dielectric constant (*ε*) of a molecular crystal can be approximated using the Clausius–Mossotti relation [44]:

(5) $$\frac{\varepsilon -1}{\varepsilon +2}=\frac{4\pi \alpha }{3V}$$

where *V* is the volume of the molecule, and *α* is the isotropic part of the molecular polarizability. Note that this expression is given in Gaussian (CGS) units. The molecular volume *V* and isotropic polarizability *α* were calculated using the Gaussian 16 program [45] at the B3LYP [46]/6‐31G [47] level of theory.

### 2.3 Koopmans Integer (KI) Functional

The calculations for IE and EA using the KI functional, a variant of the Koopmans functionals [48] that enforces Koopmans’ theorem [49] through corrections at integer electron occupations, were carried out using the Koopmans code [31]. Typically, semilocal DFT is afflicted with the self‐interaction error, which leads to inaccuracies in calculating the energy gap and related properties [26, 27, 50, 51]. The Koopmans functionals aim to address this issue by correcting the total energy of DFT, making the energy as a function of electron number piecewise linear and better aligned with physical expectations. This correction ensures that the IE and EA correspond more accurately to the negative eigenvalues of the HOMO and LUMO, respectively, fulfilling Koopmans’ theorem.

In the KI approach, the corrected total energy of the system ($E^{\mathrm{Koopmans}}$) is expressed as:

(6) $$E^{\mathrm{Koopmans}}[\rho ,\{f_{i}\},\{\alpha_{i}\}]=E^{\mathrm{DFT}}[\rho ]+\sum_{i}\alpha_{i}\left(-\int_{0}^{f_{i}}\langle \phi_{i}|{\hat{h}}^{\mathrm{DFT}}(s)|\phi_{i}\rangle ds+f_{i}\eta_{i}\right)$$

In this expression, *ρ* represents the electron density, i denotes the index of the orbitals, {*f*_i_} is the set of orbital occupation numbers, and {*α*_i_} is the set of screening parameters. The term $E^{\mathrm{DFT}}$ corresponds to the total energy computed with DFT. In this expression, $E^{\mathrm{DFT}}$ is modified by subtracting the integrated orbital energy contribution, $\int_{0}^{f_{i}}\langle \phi_{i}|{\hat{h}}^{\mathrm{DFT}}(s)|\phi_{i}\rangle \;ds$, and adding a linear correction term, *f*_i_ *η*_i_. Here, *η*_i_ represents the derivative of $E^{\mathrm{DFT}}$ with respect to the orbital occupation *f*_i_. In practice, *η*_i_ is evaluated as the difference between the total energies of the fully occupied and empty orbital configurations.

The $E^{\mathrm{DFT}}$ values were determined using the PBE [52] functional with a plane‐wave basis set and a kinetic energy cutoff of 65 Ryd, as implemented in the Quantum ESPRESSO code [53, 54]. The SG15 Optimized Norm‐Conserving Vanderbilt (ONCV) pseudopotentials [55, 56] were used to approximate the interaction between the valence electrons and the atomic core. The screening parameters ($\alpha_{i}$) [57, 58] were calculated using the ΔSCF method, which evaluates the difference in $E_{i}^{\mathrm{Koopmans}}$ for varying $\alpha_{i}$ and iteratively refines them to achieve self‐consistency [31]. The KI calculations were carried out in a nonperiodic simulation cell providing 10 Å of vacuum in each direction, ensuring that the molecule did not interact with its images.

### 2.4 GW Approximation

The GW approximation uses the Green's function [59] and the screened Coulomb interaction to compute the quasiparticle energy [29]. In the GW framework, the XC potential from DFT is replaced by the self‐energy (Σ), expressed as:

(7) Σ=iGW

where *i* is the imaginary unit, *G* denotes the Green's function which describes the propagation of quasiparticles in the system, and *W* represents the screened Coulomb interaction.

The GW calculations in this study were carried out using the BerkeleyGW code [60]. The eigenvalues and eigenvectors of the systems were calculated using the Quantum ESPRESSO DFT program [53, 54], with the PBE functional and norm‐conserving pseudopotentials obtained from the PseudoDojo library [61]. A plane‐wave basis set with a kinetic energy cutoff of 60 Ryd was employed. Quasiparticle energies were calculated using the single‐shot *G*_0_ *W*_0_ approach, and the dielectric response was modeled with the default generalized plasmon‐pole approximation [62]. The GW dielectric matrix cutoff was set to 20 Ryd.

For molecules, a *k*‐point mesh of 1 × 1 × 1 was used, while for crystals, the *k*‐point meshes were 6 × 2 × 2, 5 × 2 × 2, and 2 × 6 × 2 for PTCDA, PTCDI, and DPPDI, respectively. The number of unoccupied bands for molecules was 8859 for PTCDA, 8859 for PTCDI, and 12303 for DPPDI. For crystals, the corresponding numbers were 3318 for PTCDA (*β*‐phase), 3718 for PTCDI, and 4606 for DPPDI.

To ensure the convergence of the energy levels, the “exact_static_ch” flag was enabled. This facilitated the inclusion of a static remainder to account for the partial sum of empty bands that were excluded from the calculation due to computational limitations [63].

Since a well‐defined absolute reference is lacking for the energy levels of GW calculations, slab models with a specific surface orientation—(100) for PTCDA, (010) for PTCDI, and (010) for DPPDI—were computed. The molecules were assumed to lie flat with their larger planar surface parallel to the substrate. The energy levels were then aligned with the vacuum level set to 0, which is a standard approach applied widely in previous studies of energy level alignments [13, 64, 65].

For each crystal, a slab model was constructed to obtain the absolute vacuum level used for energy alignment. The slabs contained eight molecular layers and a vacuum region of 10 Å along the surface‐normal direction to avoid spurious interactions between periodic images. Atomic positions were kept fixed to the experimental bulk geometry, and no additional relaxation was performed. The slab was used solely to extract the planar‐averaged electrostatic potential and determine the vacuum reference; no GW calculations were performed on the slab.

### 2.5 Calculation of the First Excitation Energy

The first excitation energy of the molecules was calculated using the Q‐Chem 6.0 code. The molecular geometry was optimized with a double‐zeta cc‐pVDZ [66] basis set in conjunction with six variant XC functionals: PBE [52], PBE0 [67], BLYP [68, 69], B3LYP [46], BHHLYP [70], and B97M‐V [71]. Additionally, Grimme's DFT‐D3 [72] dispersion correction was applied. As we compared our calculations to the UV/Vis measurements conducted in solution, the C‐PCM [42] model was employed in the calculations to simulate the solvation effects. The following dielectric constants were used in the calculations: 4.0 for PTCDA crystal, 4.2 for PTCDI crystal, and 4.1 for DPPDI crystal, all derived from the Clausius–Mossotti relation, and 46.7 for dimethyl sulfoxide (DMSO).

The first excitation energy was computed using the time‐dependent density functional theory with the Tamm‐Dancoff approximation (TDDFT/TDA) [73], which simplifies the full TDDFT calculation. The same combination of the basis set and XC functionals used for geometry optimization was applied in the excitation energy calculations.

### 2.6 DPPDI Synthesis and UV/Vis

DPPDI was synthesized via the imidization of PTCDA with aniline in imidazole solvent, using zinc acetate as a catalyst. The reaction mixture was stirred for 24 h at 130°C under reflux in an argon atmosphere. After cooling, the product was precipitated with methanol, yielding a red solid, which was then washed with water and dried. Unreacted PTCDA was removed with 2% NaOH solution. Finally, the product was washed again with water and dried, resulting in a red powder.

The successful synthesis of DPPDI was confirmed through solid‐state NMR and FTIR–ATR analyses. Solid‐state NMR revealed distinct resonances consistent with the formation of imide functionalities, supporting the expected molecular structure. Complementary FTIR–ATR analysis demonstrated the disappearance of characteristic PTCDA anhydride absorption bands and the appearance of new bands attributed to the imide group.

The optical gap of DPPDI was determined using UV/Vis spectroscopy and Tauc plot analysis. UV/Vis spectra were recorded in chloroform solution from 400 to 800 nm with a Multiskan GO spectrophotometer, averaging 32 scans per measurement. The Tauc plot was constructed using the Tauc equation for an indirect gap, with *n* = 1/2:

(8) $${\left(\alpha hv\right)}^{n}=A(hv-E_{g})$$

where *α* is the absorption coefficient, *h* is Planck's constant, *v* is the frequency of the incident light, *A* is a material‐dependent proportionality constant, and *E*_g_ is the optical gap energy. The value of *E*_g_ was obtained by extrapolating the linear portion of the ${\left(\alpha hv\right)}^{n}$ versus hv plot to the energy axis, where ${\left(\alpha hv\right)}^{n}$ = 0.

## 3 Results and Discussion

### 3.1 Bridging Single Molecules and Molecular Crystals

The most direct approach to calculate the properties of molecular crystals is to perform periodic‐boundary condition (PBC) calculations using the experimental crystal structure. When such structures are unavailable, alternative strategies must be considered. One possibility is CSP, although its reliability can vary depending on molecular complexity. Another approach involves simulating the crystalline environment using implicit solvent models, such as PCM. This solvent‐based method has already been successfully applied in the calculation of properties like the energy gap and optical gap [43, 74, 75].

### 3.2 Dielectric Constant

The dielectric constants of the molecular crystal systems were calculated using the Clausius–Mossotti relation (Equation (5)). As shown in Table 1, the calculated dielectric constants (*ε*), isotropic polarizability (*α*), and volume (*V*) for PTCDA and PTCDI are almost identical, which can be attributed to the similarity in their molecular structures. However, for DPPDI, the *α* and *V* values are significantly higher than those of the other two molecules because the additional phenyl substituents increase the molecular volume and lead to more delocalized electrons, making the system more susceptible to an external electric field. The calculated dielectric constants for the three molecular crystal systems are around 4. Although the DPPDI molecule is more polarizable, it also has a larger volume, which compensates for the larger polarizability, making the *α* to *V* ratio of the three molecules similar.

**TABLE 1:** Calculated dielectric constant (*ε*), polarizability (*α*, in the unit of 10^−24^ ESU (Electrostatic Unit of Charge)), and molecular volume (*V*, cm^3^/mol).

	*ε*	*α*	*V*	*α*/*V*
PTCDA	4.0	47.9	241.2	0.199
PTCDI	4.2	49.7	243.1	0.204
DPPDI	4.1	72.4	359.7	0.201

**FIGURE 2:**
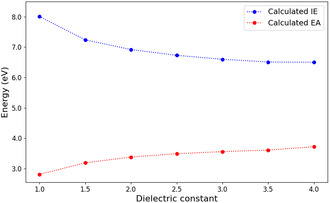
Dependence of IE and EA on the dielectric constant of the PTCDA molecular crystal system, calculated at the B3LYP [46]/6‐31G [47] level of theory.

We also investigated the influence of the dielectric constant on IE and EA within the range of 1–4, where a value of 1 represents the molecule in a vacuum, and 4 corresponds to the molecule in a molecular crystal. The correlation between the dielectric constant, IE, and EA is shown in Figure 2. As the dielectric constant increases, IE decreases while EA increases. This behavior indicates that the implicit solvent model captures electronic polarization and charge screening effects, similar to those present in a crystalline environment. These effects stabilize the charged states through electronic polarization, thereby reducing the energy gap and facilitating charge transfer. Consequently, in cases where crystal structures are unavailable, the PCM could provide a viable alternative for approximating crystal‐like polarization effects.

### 3.3 IE, EA, and the Energy Gap

Our calculated results for IE, EA, and the energy gap of isolated molecules and molecular crystals are summarized in Table 2. For isolated molecules, the calculated fundamental gap values using the KI functional and the GW approximation are in excellent agreement, while the gap predicted by LRC‐*ω*PBE is about 0.4 eV larger. For IE, the KI functional and LRC‐*ω*PBE produce similar values, whereas GW predicts a value that is approximately 0.3 eV higher. Compared to the only available experimental value—IE of the PTCDA molecule—the values computed by all three methods are generally satisfactory. It is worth mentioning that, in a benchmark study [79] of the GW100 dataset [80], which includes 100 chemically diverse small molecules, both the KI functional and GW with the PBE functional show solid performance in predicting IE, with mean absolute error (MAE) of 0.35 eV and 0.44 eV, respectively. Similarly, our results also fall within this range.

**TABLE 2:** Comparison of calculated IE, EA, and energy gap (*E*_g_) obtained using three methods (LRC‐*ω*PBE, KI, and GW) with experimental values (in eV), including mean absolute errors (MAEs) for isolated molecules (MAE_iso_) and molecular crystals (MAE_mc_) relative to experiment.

	**LRC‐** ***ω*** **PBE**			KI			GW			Exp.		
	IE	EA	*E*_g_	IE	EA	*E*_g_	IE	EA	*E*_g_	IE	EA	*E*_g_
**Isolated molecule**												
PTCDA	8.06	2.88	5.18	7.92	3.12	4.80	8.33	3.52	4.81	8.20 [76]	—	—
PTCDI	7.62	2.46	5.16	7.55	2.80	4.75	7.96	3.23	4.73	—	—	—
DPPDI	7.46	2.54	4.91	7.34	2.77	4.57	7.67	3.11	4.56	—	—	—
MAE_iso_	0.14	—	—	0.28	—	—	0.13	—	—	—	—	—
**Molecular crystal**												
PTCDA	6.50	3.72	2.77	—			7.14	4.42	2.72	6.95 [77]	4.10 [77]	2.85 [77]
PTCDI	6.25	3.35	2.90	—			6.55	3.77	2.78	6.42 [77]	4.04 [77], 3.88, 3.95 [78]	2.38 [77]
DPPDI	6.17	3.47	2.71	—			6.79	4.17	2.62	—	—	—
MAE_mc_	0.31	0.49	0.30	—			0.16	0.25	0.27	—	—	—

**FIGURE 3:**
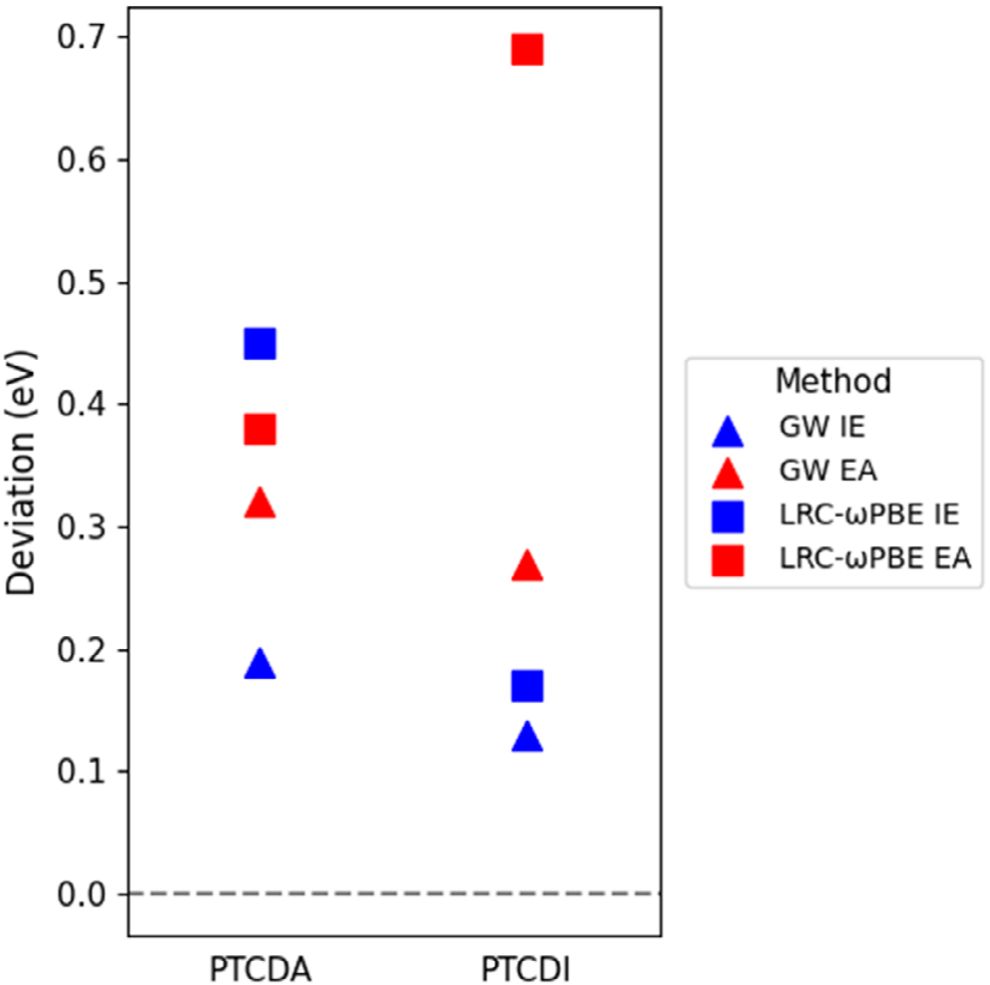
Absolute deviations of calculated IE and EA from experimental values using GW and LRC‐*ω*PBE (with PCM).

For molecular crystals, the calculated transport gap values of PTCDA using LRC‐*ω*PBE and GW reasonably match the experimental value. However, both methods overestimate the calculated transport gap value of PTCDI by 0.4–0.5 eV. As shown in Figure 3, for the calculated IE and EA, LRC‐*ω*PBE generally exhibits higher absolute deviations compared to GW. In particular, LRC‐*ω*PBE predicts the EA of PTCDI with a deviation of 0.7 eV from the experimental reference. In contrast, GW demonstrates a more accurate performance, with deviations around 0.2–0.3 eV. Moreover, it is important to note that both IE and EA are surface‐dependent [78, 81], and the magnitude of this effect due to molecular orientation at the surface for organic semiconductors can be on the order of 0.5–1.0 eV [82, 83]. In the GW calculations, we chose a specific crystallographic plane as the crystal surface, assuming the molecules lie flat and parallel to the substrate (Section 2.4). This surface effect is clearly not accounted for when using LRC‐*ω*PBE with an implicit solvent model, which simulates molecules embedded in bulk material. Additionally, the substrate on which a molecular crystal grows can influence the results, leading to variations in measured values, e.g., the experimental EA of PTCDI (Table 2).

It is important to note that the calculations for molecular crystals were performed within different theoretical frameworks: LRC‐*ω*PBE calculations were computed in a finite system using Gaussian‐type basis sets, whereas GW calculations were carried out in periodic boundary conditions with *k*‐point sampling. This makes a strictly one‐to‐one comparison between the two methods nontrivial. Besides, KI calculations for molecular crystals are not reported in Table 2. In the current implementation of the Koopmans code, the periodic workflow for KI relies on Wannierization to maximize the localization of the orbitals. For organic molecular crystals with large, highly entangled *π*/π* manifolds (such as PTCDA), obtaining a stable and chemically meaningful set of maximally localized Wannier functions can be technically challenging. As a result, although the KI approach can in principle be applied under periodic boundary conditions and has been successfully used for inorganic semiconductors and insulators [57, 84], its practical application to organic molecular crystals is still largely limited to small organic systems with relatively simple electronic manifolds [85]. An alternative strategy is provided by Koopmans‐compliant hybrid functionals, which enforce the Koopmans’ condition using localized probe states, thereby avoiding the explicit construction of Wannier functions in specific cases. Although formally distinct from KI, this approach has been successfully used to compute the energy gaps of organic molecular crystals [86].

As molecules crystallize, the energy gap value decreases significantly due to electron delocalization, which enables charge transfer with lower energy requirements. This effect is often observed in organic semiconductors and molecular solids, where closer packing leads to stronger *π–* *π* stacking and orbital overlap, thereby lowering the energy gap [87]. In addition to the delocalization effect, the reduction of the energy gap in the solid state also arises from enhanced dielectric screening provided by the surrounding molecular environment, which stabilizes charged excitations and further narrows the quasiparticle gap. Our calculations also demonstrate this trend, as the transport gap is around 2 eV smaller than the molecular fundamental gap. Furthermore, from PTCDA to PTCDI, the anhydride groups are replaced by imide groups. Since nitrogen is less electronegative than oxygen, the electrons in imide groups are more strongly pulled toward the carbonyls compared to anhydride groups, thus weakening their electron‐withdrawing ability. As a result, PTCDI has a lower IE and EA than PTCDA.

### 3.4 First Excitation Energy (the Optical Gap)

UV/Vis measurements conducted in solution yielded experimental optical gaps of 2.39 eV for PTCDA [88], 2.36 eV for PTCDI [11], and 2.29 eV for DPPDI (this work; also reported in ref. [89]). It is important to note that UV/Vis measurement conducted in dilute solution may still be affected by the solubility of the solute, potentially leading to absorption peaks arising from molecule aggregation. For instance, the appearance of charge‐transfer (CT) states, which are typically observed in aggregated systems, indicates that the molecules are not truly isolated. This behavior has been reported for PTCDA and PTCDI in the corresponding studies [11, 88]. Therefore, our calculated excitation energy refers specifically to the *S*_0_ → *S*_1_ transition of the isolated molecule.

A summary of the calculated optical gap values obtained using six variant XC functionals is presented in Table 3. Notably, the calculated values for PTCDA and PTCDI are nearly identical, while those for DPPDI are slightly smaller, following a similar trend in the calculated energy gap values. Besides, we evaluated the calculated optical gap of the isolated molecule, the molecular crystal, and the molecule in dimethyl sulfoxide (DMSO), representing three different dielectric environments with *ε* = 1, ≈4, and 46.7, respectively, using PCM. The results show that the calculated optical gap is reduced by around 0.2 eV when solvents are included, compared to solvent‐free cases. However, the gap values remain insensitive to the dielectric constant of the solvent, even as it increases from approximately 4 to 46.7. Interestingly, experimental observations also support this finding: whether in molecular thin films or in solvents with varying dielectric constants, the measured optical gap values are very similar [11, 88].

**TABLE 3:** Comparison of the calculated optical gap using variant XC functionals with experimental values (in eV). UV/Vis measurements were performed in DMSO for PTCDA, and in chloroform for PTCDI and DPPDI.

	PBE	PBE0	BLYP	**B3** **LYP**	BHHLYP	**B97** **M‐V**	Exp.
**Isolated molecule**							
PTCDA	2.45	2.76	2.43	2.69	3.06	2.60	
PTCDI	2.44	2.74	2.42	2.67	3.04	2.58	
DPPDI	2.41	2.72	2.39	2.64	3.01	2.56	
**Molecular crystal**							
PTCDA	2.29	2.59	2.26	2.51	2.89	2.43	
PTCDI	2.28	2.58	2.26	2.50	2.87	2.42	
DPPDI	2.25	2.56	2.23	2.48	2.86	2.39	
**Molecule in DMSO**							
PTCDA	2.28	2.58	2.25	2.50	2.87	2.42	
PTCDI	2.27	2.57	2.25	2.49	2.86	2.41	
DPPDI	2.25	2.55	2.23	2.47	2.85	2.39	
**UV/Vis measurements**							
PTCDA	—						2.39
PTCDI	—						2.36
DPPDI	—						2.29

**FIGURE 4:**
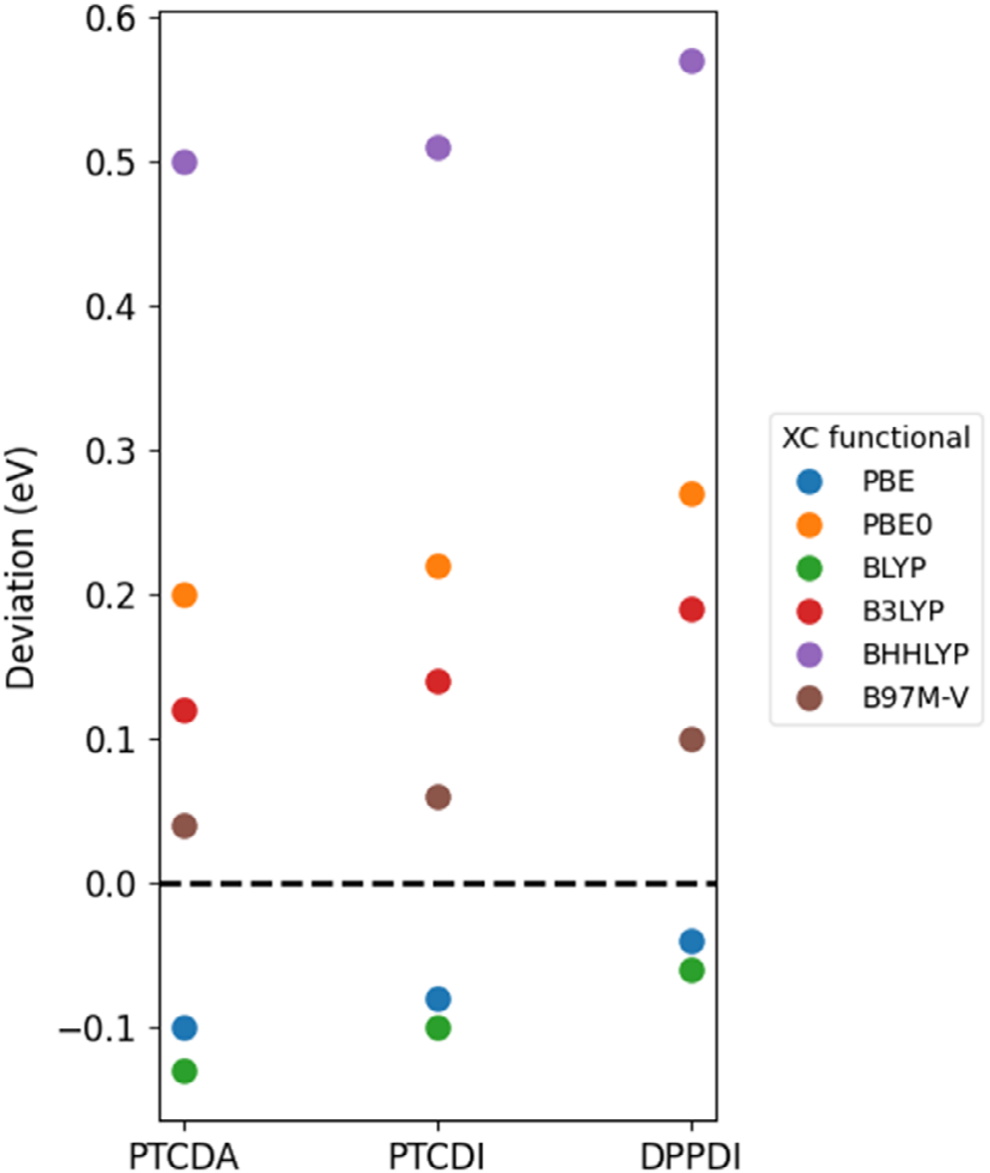
Comparison of experimental and DFT‐calculated optical gaps of PTCDA and PDI molecules using different XC functionals. The data show the deviation of the calculated results from experimental values.

We continue to discuss the influence of XC functionals. Figure 4 shows the deviations between calculated optical gaps (using the dielectric constants of molecular crystals, as the optical gaps saturate once *ε* exceeds 4) and the experimental values. PBE and B97M‐V functionals perform best in this scenario, with deviations within 0.1 eV from the experimental results. For hybrid functionals—B3LYP, PBE0, and BHHLYP, which incorporate 20%, 25%, and 50% of HF exact exchange energy, respectively—the calculated first excitation energy increases with the amount of HF exchange. This indicates that a higher proportion of HF exchange systematically leads to an overestimation of the excitation energy.

## 4 Conclusion and Outlook

We calculated the IE, EA, energy gap, and optical gap of three molecular semiconductors (PTCDA, PTCDI, and DPPDI) in both the single‐molecule and crystalline phases. To evaluate these properties, we employed a range of computational methods: RSH functionals (LRC‐*ω*PBE), the GW approximation, and the KI functional for computing the IE, EA, and energy gap; and TDDFT for computing the optical gap. For single‐molecule calculations, we applied the PCM to simulate the crystalline environment.

As a result, for molecular crystals, single‐molecule DFT with PCM is less effective in predicting IE and EA, compared to GW calculations, which utilize the actual crystal structure. However, the difference is relatively small, around 0.3–0.4 eV. This discrepancy may stem from the surface effects that the single‐molecule method does not capture. While GW calculations do not directly account for surface orientations, we employed a slab model to align the vacuum energy level to zero, thereby incorporating the effect of surface orientation. Importantly, this improved accuracy comes with a substantial increase in computational cost: GW calculations required one to two orders of magnitude more wall time than single‐molecule DFT/PCM, highlighting a practical trade‐off between precision and efficiency. This computational cost gap grows further with system size, as GW methods scale more steeply than standard DFT approaches. When the crystal structure is unavailable, the single‐molecule approach still provides quantitatively reasonable results. For computing optical gaps, single‐molecule TDDFT with PCM performs well. With a suitable choice of XC functional, such as PBE or B97M‐V, deviations from experimental measurements can be as small as 0.1 eV.

Although this study focuses on *π*‐conjugated rylene systems, the physical principles of PCM‐based TDDFT, in which localized excitations are embedded in a moderately screening environment, suggest that it should also be applicable to many other organic semiconductors. Indeed, PCM‐based TDDFT has already been successfully applied to other organic semiconductor systems [43, 74], supporting its potential generality. However, its accuracy for systems with strong CT character or pronounced anisotropic dielectric screening remains less well established [90, 91], as these features are expected to challenge TDDFT and the underlying assumptions of continuum dielectric models. Therefore, systematic comparison with experimental measurements will be important for assessing broader transferability.

Typically, finite‐system calculations struggle to accurately reproduce the properties of crystals. When calculating IE and EA in molecular systems, the PCM serves as a bridge between an isolated molecule and a molecular crystal. Although DFT calculations have been shown to reproduce the experimental dielectric constant of certain polymers [92], their accuracy for those with strongly anisotropic dielectric constants [93, 94] remains unclear. Therefore, a comprehensive benchmark comparing theoretical and experimental dielectric constants would be beneficial. Beyond this, an important question arises: can solvent‐based DFT calculations in finite systems be extended to capture other chemical/physical observables of crystals beyond energy levels and gaps? This remains a promising topic for further investigation.

## Supporting Information

Additional supporting information can be found online in the Supporting Information section. **Supporting Fig. S1:** Comparison of the experimental DPPDI structure with the predicted crystal structure. **Supporting Table S1:** Comparison of predicted and experimental DPPDI crystal structures. The lattice parameters a, b, and c are in Å; *α*, *β*, and *γ* are in degrees. The cell volume is given in Å^3^.

## Funding

This study was supported by University of Bremen and Federal State of Bremen.

## Supporting information

Supplementary Material

## Data Availability

The data that support the findings of this study are available from the corresponding author upon reasonable request.
