# Supplementary material for: Ab Initio Calculation of Energy Gap and Optical Gap of Organic Semiconductors PTCDA and PDI
Source: Chemphyschem. 2026 Feb 10;27(3):e202500481. doi: 10.1002/cphc.202500481 (PMC12890208; doi:10.1002/cphc.202500481)
Supplement: Supplementary file 1 — Supplementary Material [file CPHC-27-e202500481-s001.pdf]

ChemPhysChem

Supporting Information

Ab Initio Calculation of Energy Gap and Optical Gap of Organic Semiconductors PTCDA and PDI

Chieh-Min Hsieh[1], Tanja Link[2], Michael Maas[2], Katharina Koschek[3, 5], and Tim Neudecker[1, 4, 5]

[1] *University of Bremen, Institute for Physical and Theoretical Chemistry, Leobener Str. 6, D-28359 Bremen, Germany*

[2] *University of Bremen, Advanced Ceramics Group. Am Biologischen Garten 2 IW3, D-28359 Bremen, Germany*

[3] *Fraunhofer IFAM, Wiener Straße 12, D-28359 Bremen, Germany*

[4] *Bremen Center for Computational Materials Science, Am Fallturm 1, D-28359 Bremen, Germany*

[5] *MAPEX Center for Materials and Processes, Bibliothekstr. 1, D-28359 Bremen, Germany*

1 Computational Details: Crystal Structure Prediction

CSP was performed using the evolutionary algorithm implemented in the Universal Structure Predictor: Evolutionary Xtallography (USPEX) code.[1] Since the evolutionary algorithm requires several generations to converge, we employed a computationally cheaper method—Density Functional based Tight Binding (DFTB),[2] as implemented in the CP2K[3] code—to compute the energies of the systems. As reported by Bidault and Chaudhuri,[4] the Extended Tight Binding (xTB)[5] method combined with an evolutionary algorithm was successfully used to predict the structures of high-energy molecular crystals.

For each generation, 20 structures were generated—14 derived from the previous generation, while the remaining were generated randomly. To maintain a reasonable distance between molecules, the minimal distances between their geometric centers were set to 4 Å. For the randomly generated structures, the space group was set to $P2_1/c$, which is the most frequently occurring space group in molecular crystals (offspring structures could have symmetries different from $P2_1/c$). The geometry optimization was performed in three steps: in the first step, only the molecular geometry was optimized. In the second and third steps, the simulation cell was also optimized, with the third step employing a more rigid pressure tolerance than the second step (reduced from 200 to 100 bar).

2 Results: Crystal Structure Prediction

In our work, the experimentally observed crystal structure for PTCDI was successfully identified by the sixth generation, and for PTCDA (α-phase), by the 35th generation, through comparison with experimental crystal structures. However, the known crystal structure of DPPDI was not found after searching for more than 60 generations. Instead, a structure with a similar unit-cell volume and a slightly more condensed packing motif was predicted (Figure S1). This outcome likely reflects sampling limitations in the algorithm, which may bias the search toward more compact packing motifs. Additionally, CSP is performed effectively at 0 K and therefore neglects finite-temperature effects that may stabilize the experimental structure.

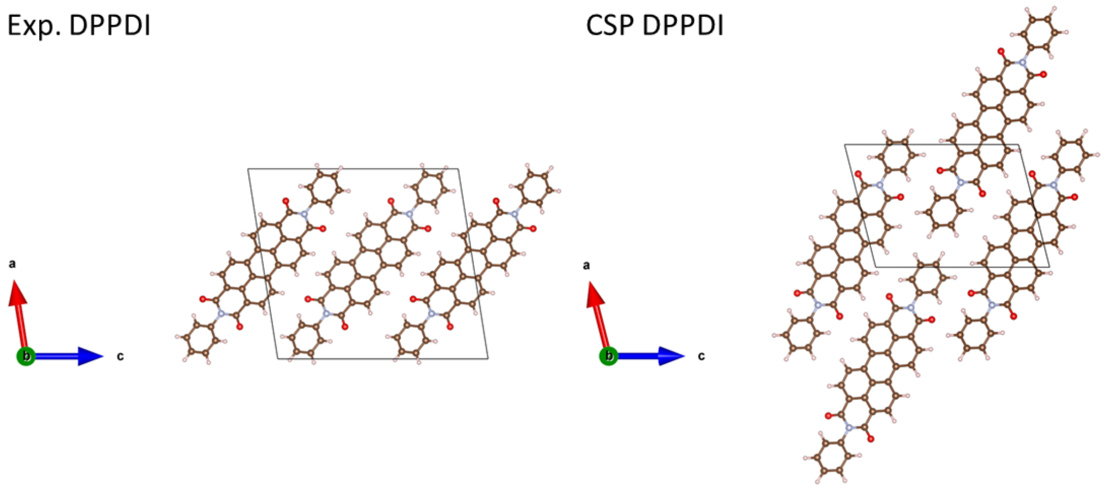

Figure S1: Comparison of the experimental DPPDI structure with the predicted crystal structure.

The main difference between the experimental and predicted DPPDI structures lies in their molecular packing—the experimental structure exhibits a higher degree of symmetry ($P2_1/c$), whereas the predicted structure is more compact and less symmetric ($P\bar{1}$). The differences in lattice parameters are summarized in Table S1. Due to the presence of phenyl groups, which introduce additional degrees of freedom in the geometry of DPPDI, molecular packing becomes in general more challenging. As the PDI molecule is functionalized with more complex functional groups, the success of crystal structure prediction using an evolutionary algorithm is expected to decrease.

Table S1: Comparison of predicted and experimental DPPDI crystal structures. The lattice parameters a, b, and c are in Å; α, β, and γ are in degrees. The cell volume is given in $Å^3$.

	a	b	c	α	β	γ	cell volume	space group
Exp.[6]	16.8	3.9	18.4	90.0	99.0	90.0	1183.8	14
CSP	10.8	7.4	15.0	79.6	103.9	89.4	1138.5	2

References

[1] Glass, C. W.; Oganov, A. R.; Hansen, N. USPEX—Evolutionary crystal structure prediction. *Computer Physics Communications* **2006**, *175*, 713–720.

[2] Porezag, D.; Frauenheim, T.; Köhler, T.; Seifert, G.; Kaschner, R. Construction of tight-binding-like potentials on the basis of density-functional theory: Application to carbon. *Physical Review B* **1995**, *51*, 12947.

[3] Hutter, J.; Iannuzzi, M.; Schiffmann, F.; VandeVondele, J. cp2k: atomistic simulations of condensed matter systems. *Wiley Interdisciplinary Reviews: Computational Molecular Science* **2014**, *4*, 15–25.

[4] Bidault, X.; Chaudhuri, S. How Accurate Can Crystal Structure Predictions Be for High-Energy Molecular Crystals? *Molecules* **2023**, *28*, 4471.

[5] Bannwarth, C.; Caldeweyher, E.; Ehlert, S.; Hansen, A.; Pracht, P.; Seibert, J.; Spicher, S.; Grimme, S. Extended tight-binding quantum chemistry methods. *Wiley Interdisciplinary Reviews: Computational Molecular Science* **2021**, *11*, e1493.

[6] Sato, K.; Mizuguchi, J. N, N-Diphenylperylene-3, 4: 9, 10-bis (dicarboximide). *Acta Crystallographica Section E: Structure Reports Online* **2006**, *62*, o5008–o5009.
